# Supplementary material for: Hybrid Models and Biological Model Reduction with PyDSTool
Source: PLoS Comput Biol. 2012 Aug 9;8(8):e1002628. doi: 10.1371/journal.pcbi.1002628 (PMC3415397; doi:10.1371/journal.pcbi.1002628)
Supplement: Text S4 — Complete source code for the PyDSTool package (version 0.88.120504). Includes API documentation and help files linking to web pages. This file is identical to the current public release on Sourceforge.net. (ZIP) [file pcbi.1002628.s004.zip › PyDSTool/html/PyDSTool.matplotlib_import-module.html]

xml version="1.0" encoding="ascii"?


PyDSTool.matplotlib\_import


| Home | Trees | Indices | Help | PyDSTool |
| --- | --- | --- | --- | --- |

Package PyDSTool ::
Module matplotlib\_import

[hide private]

[frames] | no frames]

# Module matplotlib\_import

source code

Plotting imports for PyDSTool, from Matplotlib.

Robert Clewley, March 2006.


|  |  |
| --- | --- |
| Functions | [hide private] |

|  |  |
| --- | --- |
| plot(\*args, \*\*kw) | source code |

|  |  |
| --- | --- |
| save\_fig(fignum, fname, formats=`[``'``png``'``,` `'``svg``'``,` `'``eps``'``]`)  Save figure fignum to multiple files with different formats and extensions given by the formats argument. | source code |


|  |  |
| --- | --- |
| Variables | [hide private] |

ver = `['0', '99', '0']`

failed = `False`


|  |  |
| --- | --- |
| Function Details | [hide private] |

|  |  |
| --- | --- |
| save\_fig(fignum, fname, formats=`[``'``png``'``,` `'``svg``'``,` `'``eps``'``]`) | source code |

Save figure fignum to multiple files with different formats and
extensions given by the formats argument. These are platform-dependent
and are specific to matplotlib's support.

  


| Home | Trees | Indices | Help | PyDSTool |
| --- | --- | --- | --- | --- |

|  |  |
| --- | --- |
| Generated by Epydoc 3.0.1 on Fri May 4 15:24:05 2012 | http://epydoc.sourceforge.net |
